# The effect of virtual reality on temporal bone anatomy evaluation and performance

**DOI:** 10.1007/s00405-021-07183-9

**Published:** 2021-11-27

**Authors:** Tomi Timonen, Aarno Dietz, Pia Linder, Antti Lehtimäki, Heikki Löppönen, Antti-Pekka Elomaa, Matti Iso-Mustajärvi

**Affiliations:** 1 Department of Otorhinolaryngology, Kuopio University Hospital, Puijonlaaksontie 2, 70210 Kuopio, PL 100, 70029 Kuopio, Finland; 2 Microsurgery Centre of Eastern Finland, Kuopio, Finland; 3 Department of Neurosurgery, Kuopio University Hospital, Kuopio, Finland; 4 Department of Radiology, Kuopio University Hospital, Kuopio, Finland; 5 School of Medicine, Institute of Clinical Medicine, University of Eastern Finland, Kuopio, Finland

**Keywords:** Virtual reality, Surgery training, Surgical planning, Temporal bone, Anatomy training

## Abstract

**Purpose:**

There is only limited data on the application of virtual reality (VR) for the evaluation of temporal bone anatomy. The aim of the present study was to compare the VR environment to traditional cross-sectional viewing of computed tomography images in a simulated preoperative planning setting in novice and expert surgeons.

**Methods:**

A novice (*n* = 5) and an expert group (*n* = 5), based on their otosurgery experience, were created. The participants were asked to identify 24 anatomical landmarks, perform 11 distance measurements between surgically relevant anatomical structures and 10 fiducial markers on five cadaver temporal bones in both VR environment and cross-sectional viewings in PACS interface. The data on performance time and user-experience (i.e., subjective validation) were collected.

**Results:**

The novice group made significantly more errors (*p* < 0.001) and with significantly longer performance time (*p* = 0.001) in cross-sectional viewing than the expert group. In the VR environment, there was no significant differences (errors and time) between the groups. The performance of novices improved faster in the VR. The novices showed significantly faster task performance (*p* = 0.003) and a trend towards fewer errors (*p* = 0.054) in VR compared to cross-sectional viewing. No such difference between the methods were observed in the expert group. The mean overall scores of user-experience were significantly higher for VR than cross-sectional viewing in both groups (*p* < 0.001).

**Conclusion:**

In the VR environment, novices performed the anatomical evaluation of temporal bone faster and with fewer errors than in the traditional cross-sectional viewing, which supports its efficiency for the evaluation of complex anatomy.

**Supplementary Information:**

The online version contains supplementary material available at 10.1007/s00405-021-07183-9.

## Introduction

Historically, learning of anatomy has relied on traditional methods, including two-dimensional (2D) image representations (e.g., anatomy textbooks, pictures, and radiological images), cadaver dissections and assisting or observing during live operations. Medical students, residents and young surgeons often experience difficulties in obtaining a well-grounded grasp of three-dimensional (3D) anatomy from 2D images and illustrations [1], and the inadequacy of resources for cadaver dissections and live operations may become a hindrance for adequate 3D understanding [2, 3].

Virtual reality (VR) technology offers promising stereoscopic applications for the teaching of anatomy, surgical training and preoperative planning [4, 5]. Previous studies have supported the benefits of anatomical VR visualization methods over traditional 2D methods for adding to students’ knowledge of factual anatomy [6, 7]. However, there is still a lack of clinical evidence of the impact of VR environments on learning anatomical features and achieving a contextual understanding of dimensional relationships [8–10].

Even though the new medical imaging techniques provide high resolution in each dimension, image data are still often examined as a series of 2D cross-sectional images [11]. The reduced costs, advances in computational performance and availability of consumer-grade VR technology have increased interest in medical applications of VR. It has been proposed that VR applications designed for anatomy education and surgical planning may prove themselves beneficial through the improved 3D perception of complex structures [12, 13]. In principle, VR technology can provide a 3D computer-generated environment, which make possible a stereoscopic 3D view and interaction with objects [14]. Head-mounted displays and intuitive hand-held controllers provide users with flexibility and immersion for approaching the multidimensional anatomy of the patient in ways that would otherwise be difficult or impossible.

Cross-sectional viewing or 3D reconstructions viewed from 2D screens cannot provide a similar high level of immersion and authenticity as achievable with state-of-the-art VR systems. Thus, VR may be a preferable and faster method for learning and understanding of the anatomy of the organ being investigated [15, 16]. In addition, the VR environment allows users to perceive critical anatomical landmarks and their spatial relationships in the same virtual space, which may lead to better memory recall as compared to a traditional 2D screen interface [17].

A thorough understanding of the complex anatomy of the temporal bone (TB) and the lateral skull base is a lifelong endeavor and is considered as one of the most complex anatomical regions in humans. Indeed, it poses a formidable challenge even for experienced otologic surgeons, and thus it is not surprising that novice level surgeons often experience significant difficulties in acquiring an adequate understanding of the 3D characteristics of this anatomical entity.

In a recent study, expert otologic surgeons compared the VR environment with conventional cross-sectional visualization in TBs in a simulated preoperative setting; VR was found feasible for evaluating TB anatomy with good accuracy and reproducibility [18]. The aim of the present study was to compare the VR environment to a traditional cross-sectional viewing in a simulated preoperative planning setting by either expert or novice surgeons. We investigated the impact of the viewing method on the understanding of anatomy, the accuracy of measurements and the identification of anatomical landmarks in TB anatomy. Furthermore, we studied the subjective validity and evaluated the performance of participants with the two methods in both groups.

The study’s hypothesis was that the VR environment does not offer additional benefits for the evaluation of TB anatomy compared to traditional cross-sectional (2D) viewing with equal subjective validity. In addition, we hypothesized that experienced surgeons will perform all identification and measurement tasks more accurately and faster compared to novices.

## Materials and methods

### Ethics and permissions

The study fulfilled the Helsinki Declaration for Ethical use of human material. It has an institutional approval (No. 125/2019), and the National Supervisory Authority for Welfare and Health authorized the use of cadaveric TBs (No. 9202/06.01.03.01/2013). The anonymity of participants was guaranteed, and informed consent was obtained from all participants.

### Data collection

Five fresh-frozen TBs were harvested, and five 3-mm titanium screws fiducials were placed on each TB [18]. Three of the screws placed in the outer cortex and two in petrosal part served as fixed measurement points. A standard Vernier caliper (accuracy 0.02 mm) under a surgical microscope was used to obtain the direct physical measurements (DPM) of the distances between the fiducials. TBs used in this study did not have any pre-existing pathology (e.g., malformations).

A Siemens SOMATOM Definition Flash (Siemens Healthcare, Forchheim, Germany) was used for high resolution computed tomography (HRCT) images (120 kV, 96 mAs, FOV 85 mm, pitch 0.8, CTDIvol 21.1 mGy, and 0.4 mm slice thickness). The adequacy of the HRCT quality for conventional cross-sectional viewing and VR environment, was evaluated by an experienced neuroradiologist (A.L.) and two experienced otologic surgeons (M.I-M., A.D.).

**Fig. 1:**
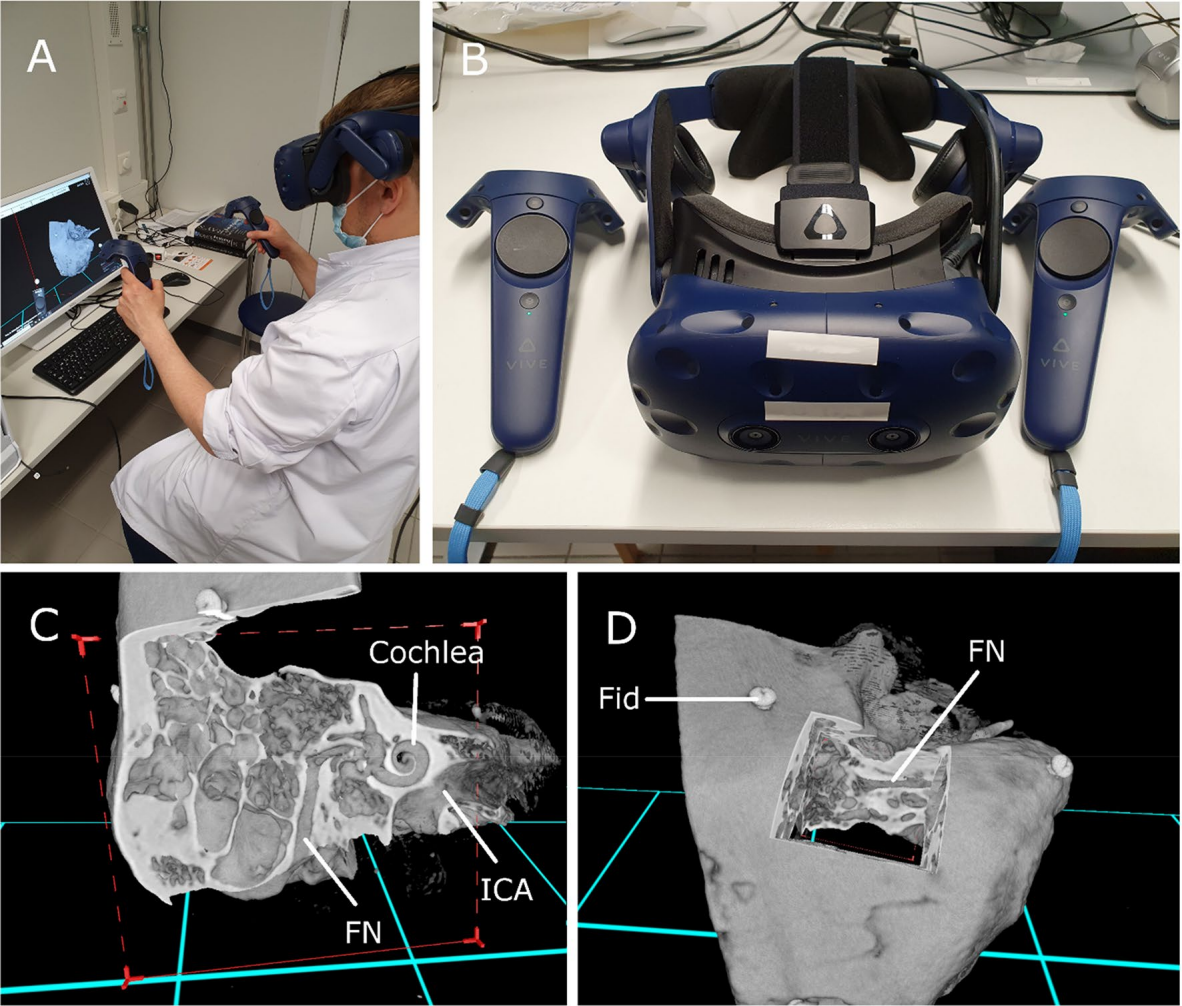
**a** Setup of the VR interface during task performance, (**b**) Head-mounted display and a pair of controllers, (**c**) and (**d**) View of a temporal bone in the VR environment. *FN* = Facial nerve, *ICA* = Internal carotid artery, *Fid* = Screw fiducial

The images were viewed as 3D volumes in the VR environment and as cross-sectional viewing in a picture archiving and communication system (PACS) interface. The 3D model from the HRCT data in the VR environment was created with the Adesante SurgeryVision™ (Adesante Oy, Turku, Finland) medical software. A head-mounted display (HTC Vive Pro, HTC, New Taipei, Taiwan) with a pair of controllers was used to visualize and manipulate the 3D models in the VR environment generated by the software (Fig. 1). The cross-sectional HRCT image visualization was performed in our clinical PACS (Sectra AB, Linkoping, Sweden) in a 2D screen interface.

### Participants

Two groups, based on their experience in otosurgery, were created: an expert group of five experienced otologic surgeons and a novice group of four residents and one neurosurgeon with no experience in TB surgery.

### Tasks

The same TBs and distance measurements as well as questionnaire data of the experts were used in our previous feasibility study [18]. In the present study, the experts were further asked to identify 24 anatomical landmarks (see Online Resource 1.). The same tasks i.e., identification of the anatomical landmarks, measuring the 11 distances between the surgically relevant anatomical structures and the 10 distances between the fiducials (see Online Resource 2.) were performed by novices for the same TBs in both VR and cross-sectional viewing. The time used by every participant for the completion of task was measured for each TB and method.

Before starting, each participant had a standardized 15-min familiarization session with the VR and PACS interfaces. The tasks were performed in a predetermined order. The five TBs were evaluated in a randomized order, first in VR and after minimum of 14 day period (mean 175d with the expert group and 170d with the novice group) in the PACS interface. This was done to minimize possible benefits for the VR environment, that could be acquired if the cross-sectional viewing was done first. The participants were allowed to adjust the image size, contrast, and brightness to their preferred setting. In addition, three preset windows optimized in the VR for bone, soft tissue and a translucent bone visualization were available. Linked multiplanar 2D reconstructions were used to make the measurements with the PACS interface. The participants were able to rotate the view and the planes in any direction to optimize the image sections for the measurements. The tasks were supervised by a senior otolaryngologist (T.T.).

### Questionnaire

After completion of the tasks, the participants evaluated the user experience and subjective validity of both methods with a modified 5-point Likert questionnaire including 20 domains (see Online Resource 3.). A free text section was added to highlight possible advantages and problems.

### Statistical analysis

All of the statistical analyses, throughout the study, were supervised by a professional statistician and performed with IBM SPSS statistics version 27 (IBM SPSS, SPSS Inc., Chicago, IL, USA). The level of significance was set to *p* < 0.05.

Inter-quartal range (IQR) was calculated for the distance measurements. Linear mixed model was used to compare the time spent in performing the tasks. The comparison of the landmark identification and measurement errors between the methods and between the groups was performed with Poisson loglinear test. Questionnaire results were compared with Wilcoxon signed rank test.

Intraclass correlation coefficient (ICC) was used as a measure of the correlation between the distances measured with different methods with the inter-rater reliability describing the agreement between different participants. Intra-rater reliability could not be tested, since the participants measured the TBs only once with each method.

The comparison of the anatomical measurements between the methods was calculated as a percentage difference to the respective median of the anatomical measurements conducted by the participants in each group.

## Results

The demographics and surgical experience are summarized in Table 1. Two novice participants had experience in cadaver TB dissections (one time for both) before task completion. Of the novices with prior experience with the VR, one had trained surgical planning once with a skull model and other had trained the use of the software, head-mounted display, and controllers on a few occasions. None of the experts had prior experience with the VR before the study.

**Table 1:** Summary of participants’ demographics. The values are medians (ranges) unless otherwise indicated

	Novice group *N* = 5	Expert group *N* = 5
Age	35 (30–40)	41 (34–61)
Years of ENT or skull base experience, *y*	4.5 (2–10)	16.5 (5.5–34)
Otological operations per year^a^	1.5 (0–6)	72 (48–120)
Sex male/female	3/2	4/1
Prior experience of VR technology^b^	2/5	0/5

^a^Total number of cases such as cochlear implant surgery, cholesteatoma, tympanoplasty, stapes surgery. Smaller operations e.g., insertion of ventilation tubes excluded

^b^Two novice participants had used VR only on a few occasions

There were some missing anatomical measurements due to a partially fractured bony ear canal in one TB. The missing measurements, five in the novice and eleven in the expert group, were excluded from the analysis. A summary of all measurements and statistics is presented in Table 2.

**Table 2:** Identification and measurement errors between (**a**) methods and (**b**) groups

A. NOVICES						
Error type	Method	Mean (SD) [%]	95% CI [%]	Mean difference [%]^a^	95% CI difference [%]^a^	*p* value^a^
Landmarks	PACS	1.43 (1.19)	0.94, 1.93	0.52	− 0.04, 1.07	0.066
	VR	0.93 (0.65)	0.67, 1.20			
Fiducials	PACS	2.32 (2.29)	1.38, 3.26	1.09	0.01, 2.17	0.049*
	VR	1.36 (2.81)	0.20, 2.52			
Anatomical	PACS	4.00 (1.96)	3.19, 4.81	0.10	− 1.40, 1.58	0.903
	VR	3.93 (2.39)	2.94, 4.91			
Total	PACS	2.26 (1.07)	1.82, 2.70	0.52	− 0.01, 1.04	0.054
	VR	1.76 (1.08)	1.32, 2.21			

A. EXPERTS						
Error type	Method	Mean (SD) [%]	95% CI [%]	Mean difference [%]^a^	95% CI difference [%]^a^	*p* value^a^
Landmark	PACS	0.77 (0.41)	0.60, 0.94	0.29	− 0.12, 0.70	0.166
	VR	0.53 (0.47)	0.34, 0.73			
Fiducials	PACS	2.08 (3.19)	0.76, 3.40	1.65	0.22, 3.09	0.024*
	VR	0.40 (0.82)	0.06, 0.74			
Anatomical	PACS	1.96 (1.81)	1.22, 2.71	− 2.36	− 3.68, -1.04	< 0.001*
	VR	4.22 (2.39)	3.23, 5.20			
Total	PACS	1.35 (0.98)	0.95, 1.76	− 0.09	− 0.53, 0.34	0.672
	VR	1.40 (0.64)	1.14, 1.67			

B. PACS						
Error type	Group	Mean (SD) [%]	95% CI [%]	Mean difference [%] ^a^	95% CI difference [%] ^a^	*p* value^a^
Landmark	Novices	1.43 (1.19)	0.94, 1.93	0.66	0.12, 1.18	0.016*
	Experts	0.77 (0.41)	0.60,0.94			
Fiducial	Novices	2.32 (2.29)	1.38,3.26	0.46	− 0.76, 0.17	0.464
	Experts	2.08 (3.19)	0.76,3.40			
Anatomical	Novices	4.00 (1.96)	3.19,4.81	2.19	0.89, 3.49	0.001*
	Experts	1.96 (1.81)	1.22,2.71			
Total	Novices	2.26 (1.07)	1.82, 2.70	0.95	0.45, 1.45	< 0.001*
	Experts	1.35 (0.98)	0.95, 1.76			

B. VR						
Error type	Group	Mean (SD) [%]	95% CI [%]	Mean difference [%]^a^	95% CI difference [%]^a^	*p* value^a^
Landmark	Novices	0.93 (0.65)	0.67, 1.20	0.40	− 0.33, 0.83	0.070
	Experts	0.53 (0.47)	0.34, 0.73			
Fiducial	Novices	1.36 (2.81)	0.20, 2.52	1.02	− 0.31, 2.34	0.011*
	Experts	0.40 (0.82)	0.06, 0.74			
Anatomical	Novices	3.93 (2.39)	2.94, 4.91	− 0.26	− 1.77, 1.25	0.734
	Experts	4.22 (2.39)	3.23, 5.20			
Total	Novices	1.76 (1.08)	1.32, 2.21	0.34	− 0.13, 0.80	0.154
	Experts	1.40 (0.64)	1.14, 1.67			

Values are proportions % unless otherwise indicated

In the landmark identification task, a misidentified anatomical landmark was classified as an identification error. The measurements outside of 1.5 IQRs were determined as outliers and counted as measurement errors in fiducial and anatomical measurements. Errors were converted into percentages per participant to allow a better comparison of task performance with each method

*VR* virtual reality environment, *PACS* conventional cross-sectional (2D) method

^a^Poisson loglinear test

*Statistically significant difference between the methods

### Landmark identification

The novice group correctly identified 95.3% and 92.8% of the landmarks in VR and cross-sectional viewing, respectively. The corresponding percentages for the expert group were 97.3% and 96.2%. Experts made significantly fewer errors in cross-sectional viewing than novices (*p* = 0.016). No statistically significant difference was found in the VR interpretation between both groups (*p* = 0.070). The comparisons and statistics between the methods and groups are presented in Table 2.

### Fiducial and anatomical measurements

A strong correlation between VR and cross-sectional viewing was found for the measurements of the fiducial (ICC_novices_ ≥ 0.906, ICC_experts_ ≥ 0.916) and the anatomical distances (ICC_novices_ ≥ 0.783, ICC_experts_ ≥ 0.900) in both groups. High inter-rater reliability (mean ICC > 0.848) was found in both cross-sectional viewing and VR methods. The mean ICCs describing inter-rater reliability of the fiducial distance measurements performed in cross-sectional viewing and VR, respectively, were 0.920 and 0.963 in the novice group and 0.930 and 0.999 in the expert group. For the anatomical distance measurements, the respective mean ICCs were 0.848 and 0.925 in the novice group and 0.914 and 0.955 in the expert group.

The statistical comparisons of the methods and groups of the measurement errors are summarized in Table 2. Twenty-two (76%) fiducial distance measurement errors were made by novices and fourteen (54%) by experts in cross-sectional viewing; these were likely due to a misidentification of the fiducial measurement points (measured distance matched to a distance between some other fiducial pair, i.e., not the correct pair). The corresponding errors in VR were 9 (53%) and 0 in the novice and expert group, respectively.

### Errors in total

Statistical analysis of all errors revealed that the novice group made significantly more errors with cross-sectional viewing than the expert group (127 errors vs. 76 errors, *p* < 0.001), whereas with VR, the difference between the groups remained statistically insignificant (Table 2). In total, the novices made fewer errors with VR than with cross-sectional viewing (99 errors vs. 127 errors, *p* = 0.054). In the expert group, the number of errors was not method dependent (*p* = 0.672).

**Fig. 2:**
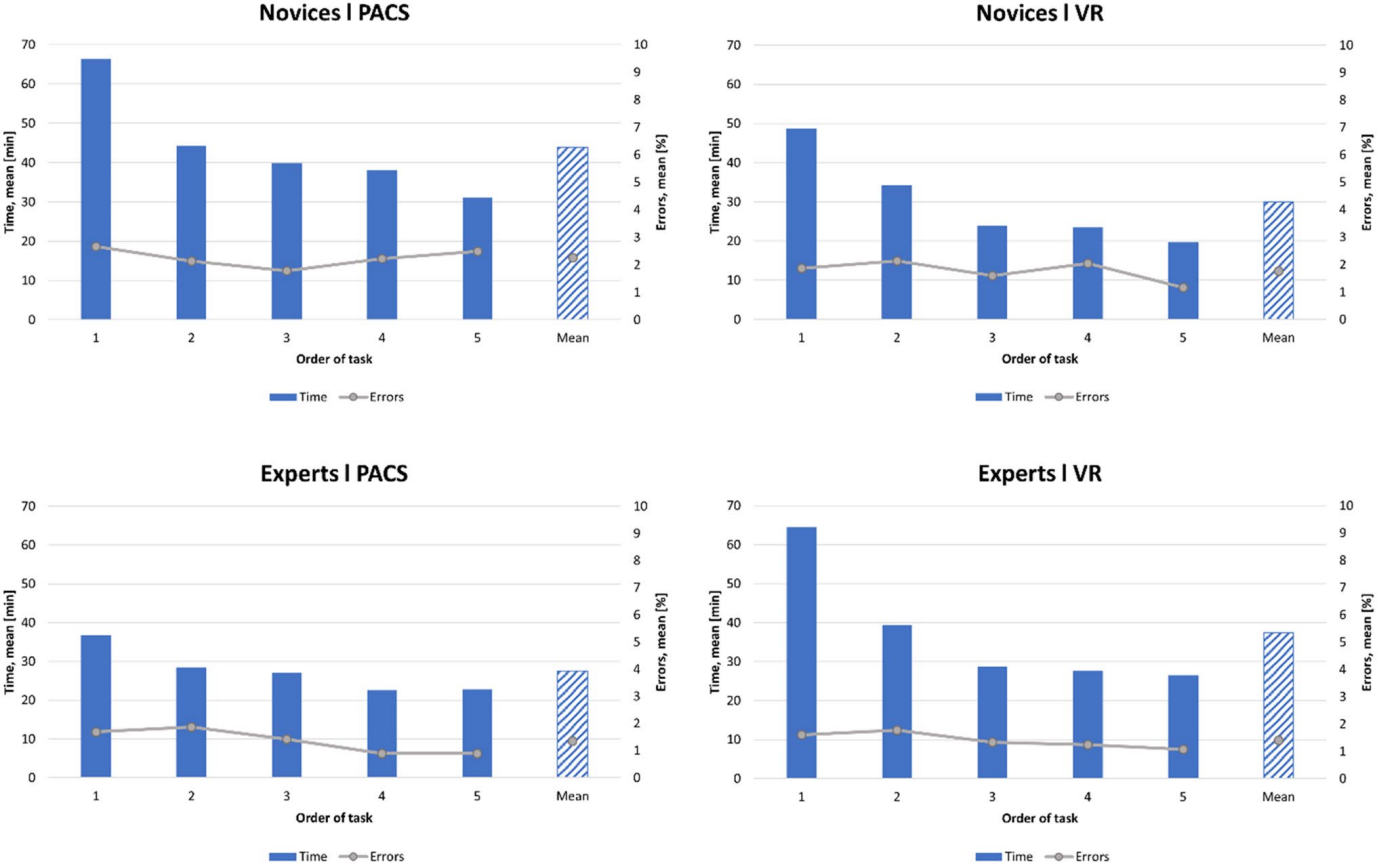
Mean time used and number of errors (%) with TB task progression in both study groups

When the first TB task was compared to the last, the number of errors in total were found to decrease in both groups with both methods. In the expert group, the mean difference of errors between first and last TB was 2.67% (*p* = 0.273) in VR and 4.00% (*p* = 0.095) in cross-sectional viewing. In the novice group, the corresponding values were 3.56% (*p* = 0.170) in VR and 0.89% (*p* = 0.793) in cross-sectional viewing. A summary of the overall error development is presented in Fig. 2.

### Time needed for task completion

The statistics and development of time used for task completion are illustrated in Fig. 2; Table 3. The experts were significantly faster when using the cross-sectional viewing in PACS interface than novices, but with VR, the difference between the groups remained statistically insignificant.

**Table 3:** Duration of tasks between methods and groups. All values are times (min) unless otherwise indicated

	Method/Group	Mean (SD) [min]	95% CI [min]	Mean difference [min]^a^	95% CI mean difference [min]^a^	*p* value^a^
Novices	PACS	43.89 (21.86)	37.04, 50.74	13.85	4.96, 22.74	0.003*
	VR	30.04 (15.17)	6.68, 53.39			
Experts	PACS	27.53 (11.79)	20.68, 34.38	− 9.81	− 17.72, − 1.90	0.016*
	VR	37.34 (18.43)	13.98, 60.67			
PACS	NOVICES	43.89 (21.86)	28.68, 59.09	16.36	6.64, 26.04	0.001*
	EXPERTS	27.53 (11.79)	16.99, 38.07			
VR	NOVICES	30.04 (15.17)	14.83, 45.24	− 7.03	− 40.34, 25.73	0.665
	EXPERTS	37.34 (18.43)	26.80, 47.88			

*VR* = virtual reality environment, *PACS* = conventional cross-sectional (2D) method

^a^Linear mixed model test

*Statistically significant difference between the methods

In the comparison of the time development between the first and the last TB task, the mean difference of time (minutes) used in novice group was 35.27 in cross-sectional viewing (*p* < 0.001) and 29.12 in VR (*p* < 0.001). In the expert group, the corresponding values were 14.00 (*p* = 0.009) and 37.99 (*p* < 0.001).

### Questionnaire

The scores for the questionnaire are illustrated in Table 4. The mean overall scores for VR were significantly better than those for cross-sectional viewing in both novice and expert groups (*p* < 0.001).

**Table 4:** Responses to the questionnaire

	Novices		*p* value^a^	Experts		*p* value^a^
	VR (SD)	PACS (SD)		VR (SD)	PACS (SD)	
Appearance of anatomical structures	4.6 (0.5)	3.2 (0.4)	0.059	4.6 (0.5)	3.4 (0.5)	0.034*
Appearance of tools	3.4 (0.5)	3.6 (1.0)	0.785	3.2 (0.4)	3.2 (0.7)	1.000
Usability of tools	3.4 (0.8)	3.2 (0.7)	0.705	3.0 (0.6)	3.0 (1.1)	1.000
Performance of tools	4.0 (0.6)	3.4 (1.2)	0.334	3.8 (0.4)	3.4 (0.5)	0.157
Haptic feedback	3.2 (1.3)	2.4 (1.2)	0.414	3.6 (0.5)	3.0 (0.6)	0.830
Ergonomics	4.0 (0.9)	3.6 (0.5)	0.414	3.2 (0.4)	3.6 (1.0)	0.317
Depth perception	4.6 (0.8)	2.0 (0.6)	0.059	4.4 (0.8)	2.0 (0.9)	0.034*
Quality of graphics	4.2 (0.7)	3.2 (0.7)	0.180	4.4 (0.8)	4.0 (0.9)	0.317
Learning of anatomy	4.8 (0.4)	3.2 (0.7)	0.039*	4.6 (0.5)	3.2 (0.4)	0.059
Learning of surgical planning	4.6 (0.8)	2.8 (1.2)	0.066	4.6 (0.5)	3.4 (0.5)	0.063
Understanding of anatomical structures	4.8 (0.4)	3.2 (1.2)	0.034*	4.4 (0.5)	3.4 (0.5)	0.034*
Quality of measuring anatomical structures	4.4 (0.5)	3.2 (1.5)	0.336	4.4 (0.5)	2.8 (0.7)	0.059
Understanding the relationships of anatomical structures	4.8 (0.4)	2.8 (1.0)	0.041*	4.6 (0.5)	3.0 (0.6)	0.180
Accuracy of measurement tool	4.4 (0.8)	4.0 (0.9)	0.414	4.0 (0.9)	3.6 (0.8)	0.577
Hand–eye-coordination	4.4 (0.8)	3.2 (1.0)	0.141	3.8 (0.4)	3.0 (0.0)	0.046*
Overall score for surgical planning	4.4 (0.5)	3.2 (1.0)	0.109	4.2 (0.4)	3.4 (0.5)	0.102
Global rating						
Recommend to colleague	4.6 (0.5)	3.8 (1.5)	0.414	4.6 (0.5)	4.2 (0.7)	0.157
User-friendly	4.4 (0.5)	3.0 (1.1)	0.059	3.6 (0.8)	3.6 (1.0)	1.000
Inclusion to surgical planning	4.6 (0.5)	4.0 (1.1)	0.414	4.6 (0.5)	4.8 (0.4)	0.317
Understanding of the surgical site	4.8 (0.4)	3.4 (1.0)	0.066	4.6 (0.5)	3.8 (0.4)	0.046*
Mean overall scores	4.3 (0.8)	3.2 (1.1)	< 0.001*	4.1 (0.8)	3.4 (0.9)	< 0.001*

All values are nominal (SD) on a 5-point Likert scale unless otherwise indicated. Score 1 represented not true/realistic/useful and 5 represented very true/realistic/useful. A score of 3 was considered neutral

*VR* = virtual reality environment, *PACS* = conventional cross-sectional (2D) method, *SD* = standard deviation

^a^Wilcoxon signed rank test

*Statistically significant difference between the methods

An analysis of the free text feedback revealed that VR provided a better 3D understanding of the anatomical structures and their relationship. The freedom to approach the target from any direction and angle in the VR environment was much appreciated. All participants rated the VR as an excellent tool for learning anatomy and for preoperative planning.

## Discussion

Previous studies have demonstrated the feasibility and effectiveness of exploiting 3D techniques and VR environments in teaching anatomy, especially for medical students [19–21]. For example, in reconstructive surgery, there are several studies demonstrating that virtual surgical planning may improve surgical accuracy and clinical outcomes [22–24]. Studies focusing on TB anatomy have evaluated the feasibility of VR for different simulator platforms (i.e., mastoidectomy training) targeted for surgical training and skill assessment [25, 26]. To the best of our knowledge, there are only a few studies that have investigated the effect of VR technology on learning the anatomy of TB in residents and experienced otosurgeons [27, 28]. Therefore, we felt it would be interesting to examine the impact of VR for anatomy understanding and on performance in different anatomical tasks in a simulated preoperative planning setting in TBs.

Previously, it has been demonstrated that in experienced surgeons, the VR environment can provide equally accurate results as compared to conventional cross-sectional viewing [18]. Our present study explored the effect of a VR environment designed for surgery planning with respect to measurement accuracy, identification of anatomical structures, participants performance evaluating TB anatomy, and subjective validity of the TB’s complex anatomy in novice and experienced surgeons.

We detected strong correlations between all distance measurements for each method in the novice group supporting the validity of measurements in VR. The time needed to complete the tasks and the numbers of errors demonstrated that the expert group was significantly faster and more accurate than the novice group when making the measurements with the cross-sectional views. In addition, this study revealed a rather long learning curve of the novice participants with cross-sectional viewing of complex anatomical structures compared to that in the VR. Our results demonstrate that two-dimensional images are appropriate and adequate for pre-operative planning for surgeons with prior experience and pre-existing anatomic knowledge gained from continuous surgical training and clinical work. In addition, expert surgeons are also trained to use cross-sectional viewing with PACS interface in preoperative planning and, therefore, possess better skills and ability to mentally reconstruct the cross-sectional data into a complex 3D model. For novices, however, our results show that VR viewing may convey additional information, which they can obviously utilize for a faster apprehension of the complex TB anatomy.

Interestingly, the 3D model readily created in the VR environment appeared to provide a better foundation for understanding complex anatomical structures, such as the TB, since the VR method may not require as much mental 3D reconstruction experience as the cross-sectional viewing with PACS. This argument can be supported by the following findings: with the VR, the novices had a lower number of errors for every task (landmark identifications, fiducial, and anatomical measurements) and significantly better time consumption results as compared to the cross-sectional viewing. A rather compelling finding was that there were no significant differences between experts and novices in time consumption or the number of errors in VR environment and thus refuting our second hypothesis. This is of special importance considering the fact that the difference of errors in the expert group remained insignificant between VR and cross-sectional viewing. In addition, with VR, the total number of errors decreased more in the novice than in the expert group, and the novices’ results in VR moved closer to the expert level as the tasks progressed from first to fifth TB, indicating a potentially shorter learning curve as compared to cross-sectional viewing. These findings demonstrate that VR may be advantageous especially for novice surgeons to acquire an in-depth 3D understanding of complex anatomical structure.

The results of subjective validity contradict with our hypothesis, as both groups favored the VR environment over cross-sectional viewing. This result may be due to the authenticity that was provided by VR. The results also demonstrate the surgeons’ positive attitude for adopting VR into anatomy learning, training and preoperative use irrespective of their level of experience, which is consistent with previous VR simulator studies found in literature [25, 29]. The novices rated VR higher (and cross-sectional viewing lower) than the expert group. This may reflect the fact that they are more dependent on the additional information provided by VR. In addition, they may be more interested in exploiting new technologies in their training and clinical use in comparison to experienced surgeons. However, the experts also favored VR over cross-sectional viewing.

Several studies have demonstrated that there is extensive variability in the spatial comprehension of individuals, which may affect learning of anatomy and eventually their abilities to perform the kinds of tasks demanded in this study [30, 31]. Since both groups had one participant that made a substantial amount of incorrect fiducial measurements, it is assumed that this applies also to the participants of this study. However, it has also been demonstrated that visual 3D models could improve learning of anatomy in individuals with poor spatial abilities [32].

Although, we used the term “learning” on several occasions, we are aware of the fact that learning is a broader concept and thus not completely accurate, since this study focused on the evaluation of a novel system for presenting complex anatomy. However, for novices we observed improvements in performance with VR (less errors in less time), which represented undoubtedly a learning effect. Therefore, we think, the intuitive 3D representation of VR may enhance also learning.

### Limitations

This study has certain limitations that require attention. The number of participants was small. With respect to the anatomical identifications and measurements, the large number of measurements points (1125 per group) ensured adequate statistical power. Due to the high inter-rater reliability, it can be argued that a larger number of participants would not have changed the results. Although it has been shown that there is a considerable individual variety in spatial comprehension abilities, it was beyond the scope of our study to test each participant’s talents. However, including these individual skills in future trials would be beneficial to obtain a more detailed evaluation of the role of VR in anatomy learning and operative planning. Furthermore, the impact of VR technology on the surgical performance and clinical outcomes as well as surgery training (e.g., mastoidectomy training) was not investigated here and will be a topic to be evaluated in the future.

## Conclusion

In the VR environment, novices were faster and committed fewer errors than in traditional cross-sectional viewing. In addition, novices´ task performance approached the level of expert otosurgeons after only five TB VR sessions, indicating a potentially shorter learning curve for novices in evaluating temporal bone anatomy compared to cross-sectional viewing and supporting efficiency of the VR environment for evaluating complex TB anatomy. For experts, no difference in task performance between VR and cross-sectional viewing was found. Surgical experience of experts may effectively compensate the more limited information of cross-sectional viewing; however, as novices, they also appeared to appreciate the availability of a 3D representation of TB anatomy.

## Supplementary Information

Below is the link to the electronic supplementary material.

Supplementary file1 (PDF 88 KB)

Supplementary file2 (PDF 54 KB)

Supplementary file3 (DOCX 18 KB)

## Data Availability

The datasets used and/or analysed during the current study are available from the corresponding author on reasonable request.
