# Supplementary material for: The effect of virtual reality on temporal bone anatomy evaluation and performance
Source: Eur Arch Otorhinolaryngol. 2021 Nov 27;279(9):4303–12. doi: 10.1007/s00405-021-07183-9 (PMC9363303; doi:10.1007/s00405-021-07183-9)
Supplement: Supplementary file 1 — Supplementary file1 (PDF 88 KB) [file 405_2021_7183_MOESM1_ESM.pdf]

Supporting information 1. List of anatomical landmarks in the identification task

Anatomical landmark	
Carotid artery	Oval window
Sigmoid sinus	Internal acoustic meatus
Jugular bulb	Lateral semicircular canal (prominence)
Tympanic tegmen/dura lamel	Superior semicircular canal
Sinodural angle	Posterior semicircular canal
Facial nerve (mastoid/vertical)	Vestibulum (utricule)
Facial nerve (tympanic/horizontal)	Umbo
Geniculate ganglion	Corpus of incus
Facial nerve (labyrinthine)	Incudomalleolar joint
Facial nerve (meatal)	Scutum
Cochlea apex	Cochlear aqueduct
Round window	Vestibular aqueduct (saccus)
