# Supplementary material for: The effect of virtual reality on temporal bone anatomy evaluation and performance
Source: Eur Arch Otorhinolaryngol. 2021 Nov 27;279(9):4303–12. doi: 10.1007/s00405-021-07183-9 (PMC9363303; doi:10.1007/s00405-021-07183-9)
Supplement: Supplementary file 2 — Supplementary file2 (PDF 54 KB) [file 405_2021_7183_MOESM2_ESM.pdf]

Supporting information 2. Measured anatomical distances and their clinical relevance.

Anatomical measurements:	Indication for measured distance:
Length of malleus (manubrium+head)	Universal measurement, easy to compare, no direct surgical relevance.
Distance from body of incus to mastoid cortex	The estimate for drilling depth, e.g. in mastoidectomy.
Horizontal diameter of bony ear canal (external meatus)	Estimation of drilling work during canaloplasty.
Vertical diameter of bony ear canal (internal meatus)	Estimation of tympanic membrane diameter, universal anatomic measurement.
Size of facial recess	Space for posterior tympanotomy.
Distance from facial recess to mastoid cortex	The estimate for drilling depth regarding the facial nerve.
Distance from facial nerve (mastoid part) to recess of bony ear canal	Limit of posterior drilling in canaloplasty.
Diameter of oval window	Stapedotomy, stapedectomy. Identification of the oval window area and surgical access to stapes footplate.
Diameter of round window	Cochlear implantation and active middle ear implantation. Identification of round window area and evaluation of surgical approach.
Length of styloid process	Universal anatomical measurement, stylalgia.
Distance from sigmoid sinus to back wall of bony ear canal	The space available for drilling in e.g. mastoidectomy.
