# Supplementary material for: The effect of virtual reality on temporal bone anatomy evaluation and performance
Source: Eur Arch Otorhinolaryngol. 2021 Nov 27;279(9):4303–12. doi: 10.1007/s00405-021-07183-9 (PMC9363303; doi:10.1007/s00405-021-07183-9)
Supplement: Supplementary file 3 — Supplementary file3 (DOCX 18 KB) [file 405_2021_7183_MOESM3_ESM.docx]

Supporting information 3. User-experience questionnaire for cross-sectional image viewing (PACS) and VR environment (VR)

Grading scale 1-5

1. *not true/realistic/useful*
2. *somewhat not true / realistic / useful*
3. *neutral*
4. *somewhat true / realistic / useful*
5. *very true / realistic / useful*

|  | PACS | VR |
| --- | --- | --- |
| Appearance of anatomical structures |  |  |
| Appearance of tools |  |  |
| Usability of tools |  |  |
| Performance of tools |  |  |
| Haptic feedback |  |  |
| Ergonomics |  |  |
| Depth perception |  |  |
| Quality of graphics |  |  |
|  |  |  |
| Learning of anatomy |  |  |
| Learning of surgical planning |  |  |
| Understanding of anatomical structures |  |  |
| Quality of measuring anatomical structures |  |  |
| Understanding the relationships of anatomical structures |  |  |
| Accuracy of measurement tool |  |  |
| Hand-eye-coordination |  |  |
| Overall score for surgical planning |  |  |
|  |  |  |
| Global rating: |  |  |
| Recommend to colleague |  |  |
| User-friendly |  |  |
| Inclusion to surgical planning |  |  |
| Understanding of the surgical site |  |  |

**Free response section:**

1. Did you experience nausea/vertigo/headache or any other similar problems during the use of VR application?
2. What kind of problems did you experineced in VR surgical planning? And in PACS method?
3. What would be an adequate time for orientation of the VR equipment?
4. What kind of benefits you consider the VR environment has compared to PACS method in surgical planning and in the understanding of topographical anatomy of objects?
5. In what other applications VR environment could be applied (e.g. education/surgical training)
6. Free feedback
